# Wood pellets transport with vibrating conveyor: experimental for DEM simulations analysis

**DOI:** 10.1038/s41598-021-96111-2

**Published:** 2021-08-16

**Authors:** Daniel Gelnar, Rostislav Prokeš, Lucie Jezerska, Jiri Zegzulka

**Affiliations:** 1 CEET, ENET Centre, Bulk Solids Centre, VSB-TU Ostrava, 17. listopadu 15, 70800 Ostrava, Czech Republic; 2 Department of Mining Engineering and Safety, Faculty of Mining and Geology, VSB-TU Ostrava, 17. listopadu 15, 70800 Ostrava, Czech Republic

**Keywords:** Chemical engineering, Mechanical engineering

## Abstract

This work presents a comprehensive overview of the mechanical-physical parameters of the transport material affecting the vibratory transport. For this purpose, spruce pellets of different lengths, oak rods and spruce crush were tested. The determined parameters were particle size distribution and shape, internal friction, static and dynamic angle of repose. The samples were transported by a patented validation vibrating conveyor. Various settings were used. The results show that by changing the shape, it is possible to reduce friction or resistance as well as energy intensity during transport. It was observed that perfect shapes and lighter particles have lower friction, but a more pronounced bounce. Therefore, it does not form a typical pattern during transport, as in the case of an imperfectly shaped one. There is also included a simulation of the discrete element method. The study shows the possibility of the vibration machine where the material can be conveyed either directionally or sorted.

## Introduction

Vibrating transport is widely used in various industries from the chemical to the construction industry. During vibrational transport, various phenomena occur, such as the formation of flow profiles/models/patterns (pattern formation)^1,2^, size or density segregation^3–5^, surface waves, arches, or fluidisation^6^.

Pattern formation is the result of a dynamic process where the particles are arranged in the form of strips. This stratification generally occurs in granular systems^1,7^. A well-known example is a mixture of small and large particles, where different shapes of piles are formed during pouring, which is due to the different angles of repose of particles studied^8,9^. Krengel^1^, based on Molecular Dynamics Simulations, states that friction is responsible for the formation of samples, i.e. tangential contact interaction between two particles as well as between the particles and the contact material conveyor. If the particles are in contact with the material of the conveyor and at the same time in contact with another particle, it is impossible to roll the particle over the contact material of the conveyor. This phenomenon causes local jamming/local interference/clusters and eventually the formation of a strike-like pattern. These stripes oriented perpendicular to the direction of vibration, are unstable and may combine or crumble. The pattern formation process can have several phases depending on the parameters of the equipment used, the degree of filling and, last, but not least, the properties of the tested material^10^. Ciamara^11,12^ describes stripe formation as a phenomenologically monodisperse system of particles interacting through an anisotropic short-range interaction. It states that granular mixtures subject to horizontal vibrations are also subject to mutual interaction forces, the basic characteristic of which is directional anisotropy. Another significant phenomenon observed in pattern formation, especially in the cluster state, is that heavier particles are characterised by larger fluctuations in kinetic energy^13^. The basic mechanism is still not sufficiently understood.

The method, quality, performance of transport and storage of bulk materials can be affected by parameters such as shape, symmetry, size of transported particles^14^ and moisture^15^. Less examined properties that can also affect the transport of vibrational particles are friction parameters^16,17^. Evidently, when vibrations are applied, the behaviour of the particle changes. Previous studies have shown that the rheological behaviour of powders is altered, for example, by local blockage due to the size of the particles and therefore their flow is stopped^18–20^. In particular vibrations modify the deposits by permitting grain reorganisations close to the flow arrest^21^.

With the rapid development of computer technology, the Discrete Element Method (DEM) has become a powerful tool for understanding the principles of transportation systems. There are many works devoted to DEM creation/calibration/validation of models studying vibration systems^14,22–26^. They deal with the influence of vibration amplitude and vibration frequency on the degree of particle segregation^22^ or on flow dynamics^23^. Others show the determination of DEM input parameters for non-spherical particles and their influence on the final model, along with the influence of sphericity and particle size distribution on velocity and motion characteristics of particles^14^.

Knowledge of the mechanical-physical properties of the transported material is important for the design and efficient operation of the equipment used. Their mechanical-physical properties can be used as a scale of quality or for the possibility of process prediction. The aim of the work is, therefore, to analyse in detail the influence of mechanical-physical properties of the material and the parameterisation of the device on the method of vibration transport as well as the consequent influence of the transport time on a straight track or on a track with passive elements. Based on the determined parameters, a DEM (Discrete Element Method) model was created, which can be a significant help in the overall analysis of the process of vibrational transport and the creation of structures of tested wood pellets after the subsequent validation of the simulation by real experiments^26^.

## Materials, methods and validation vibrating conveyor

### Materials

**Figure 1:**
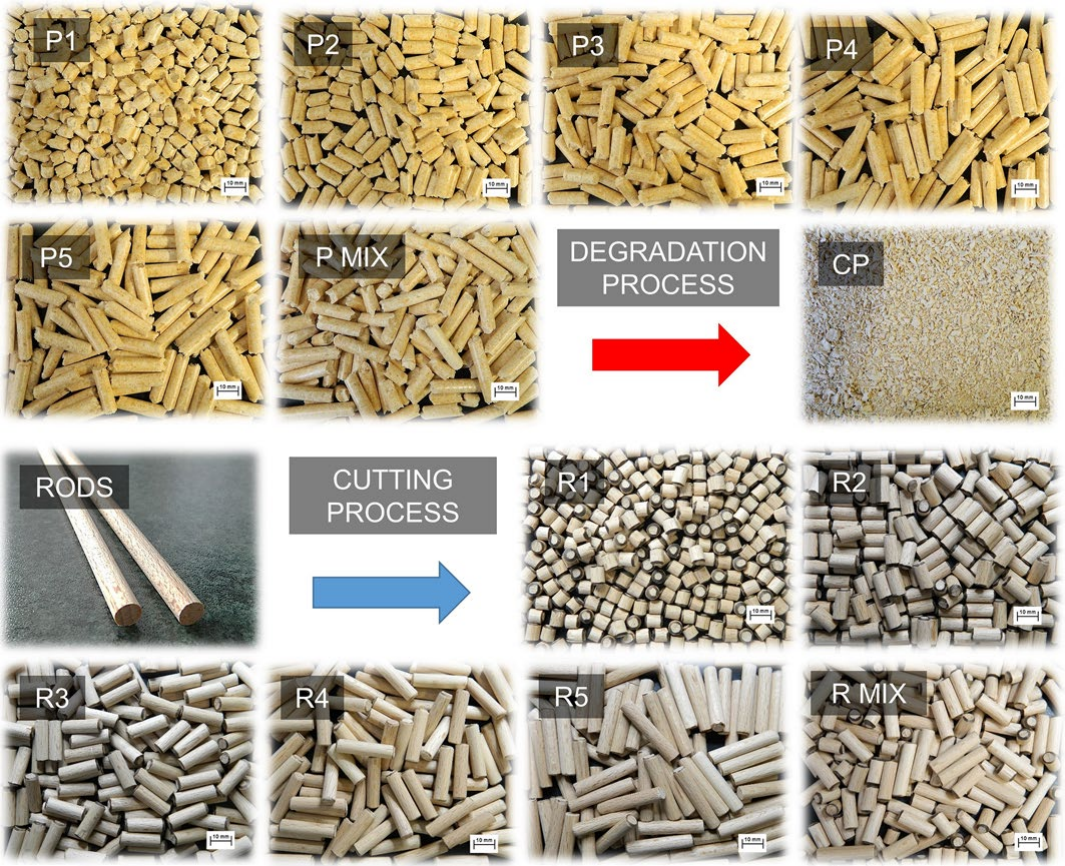
Particles used in experiments.

Pellets from spruce wood with a diameter of 6 mm were used in this study. These are commercially available pellets designated as Top A1 from Biomac Ltd. The use of wood pellets in the EU is expected to grow in sectors such as co-firing in coal-fired power plants and residential heating in the short term and in the form of high quality industrial heat in the long term^27^. The pellets are of various lengths in the range of 5–40 mm. For the experiments, the pellets were separated into size classes of length shown in Fig. 1. Samples of the pellets were designated as P1 (6 mm), P2 (12 mm), P3 (18 mm), P4 (24 mm) and P5 (30 mm). P MIX represents a real mixture of spruce pellets. The last sample was crushed pellets (CP).

The “ideal shape and firmness” solid wooden rod has been used as a reference sample for spruce pellets, which are not perfect in shape and firmness. Wooden bars of 6 mm diameter are commercially available from Hornbach Baumarkt CS Ltd. The rods were cut for comparison to lengths equal to those of spruce pellets, and the edges were trimmed. The markings were R1 (6 mm), R2 (12 mm), R3 (18 mm), R4 (24 mm) and R5 (30 mm). R MIX represents an analogous mixture to a real spruce pellet mixture (Fig. 1). The density of spruce pellets is 1172 kg/m^3^, spruce crushed pellets 182 kg/m^3^ and wooden oak rods 733 kg/m^3^.

### Methods

#### Particle shape and particle size distribution

**Figure 2:**
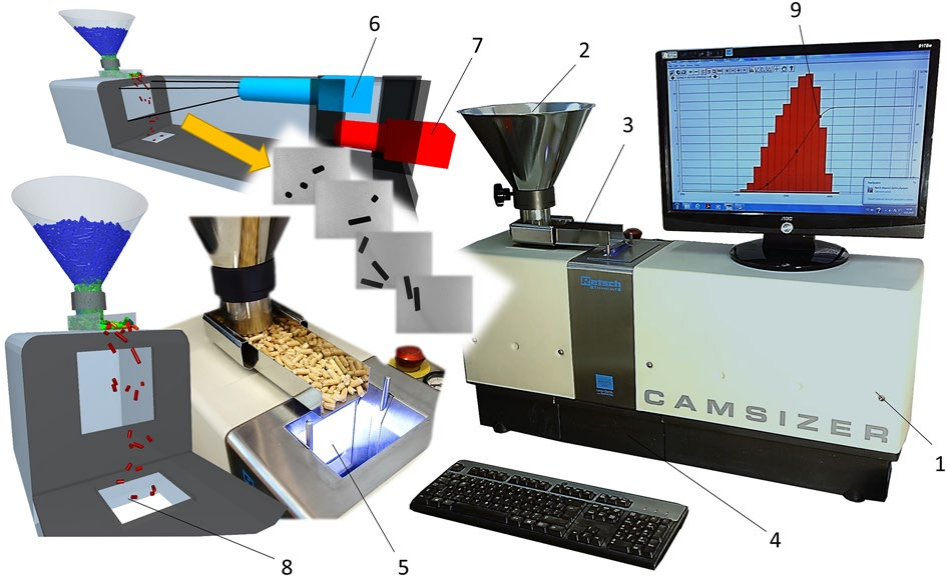
Camera particle analyser—CAMSIZER. 1—device cover, 2—filling funnel, 3—vibrating feeder, 4—catch vessel, 5—illuminated measuring cell, 6—CCD camera zoom 1, 7—normal CCD camera 2, 8—exit hole, 9—PC with software.

The microscopic quantification of the shape of the samples tested (crushed pellets, pellets, and rods) was determined using the Expert Shape Image Analysis System, which is part of the Cilas PSA 1190 facility (Anton Paar, Les Ulis, France). The dry samples were taken by the Camsizer with integrated CCD cameras (Fig. 2) for both particle size distribution and particle shape sensing. The photographs were electronically uploaded to Expert Shape. The following basic shape parameters were determined: sphericity, circularity, elongation, and convexity. Table 1 shows the basic geometric parameters and calculation formulas used in this study.

**Table 1:** Table of shape parameters used in the study.

Shape parameter	Formula	Description
Sphericity	R_circum inscribing_/R_circum scribing_	Diameter of inscribed circle by diameter of scribed circle
Circularity	4πA/p^2^	A is the area of the particle, and p is its circumference
Elongation	1 − Aspect ratio	
Aspect ratio	L_Feret’s min_/L_Feret’s max_	Length of Feret’s minimum by length of Feret’s maximum
Convexity	P/P_convex_	Perimeter (P) by convex perimeter of particle (P_convex_)

The measured materials were thus inserted into a filling funnel (2) from which they were dosed by a vibrating feeder (3) into an illuminated measuring cell (5). The gradual speed of this conveyor traffic is set by a program that automatically controls vibrations and thus also the amount of falling particles in front of the CCD camera zoom (6), along with the normal CCD camera (7), so that they do not overlap with each other. Subsequently, the material fell through the exit hole (8) into the catch vessel (4), from which it was then refilled into the funnel, and the measurement was repeated. Each sample was measured 10 times.

Sphericity is a measure of the degree to which the shape of a particle approximates that of a true sphere^28,29^. Circularity is a measure of how closely a particle boundary approximates to a circle^30,31^. Elongation is the measure of the particle’s extension. The extension values are in the range of 0–1. The symmetric shape of the particles in all directions, such as a circle, shows an elongation of 0, while a shape with different aspect ratios will have an elongation closer to 1. The aspect ratio underlying elongation is defined as the ratio of the Feret’s minimum length to the Feret’s maximum length and has been used historically to classify the general form of particles (e.g., equant, acicular, or fibrous)^32^. The convexity is a measurement of the particle edge roughness and the particle compactness and is defined as the ratio of the actual projection area and the convex hull area^32^.

#### Angle of internal friction

**Figure 3:**
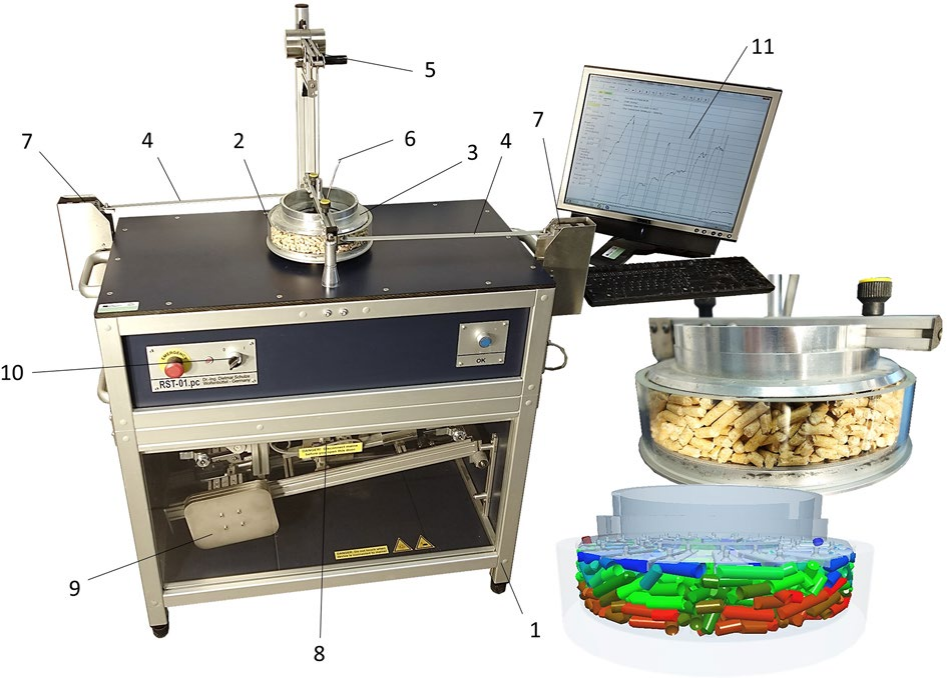
Schulze ring shear tester RST-01—machine frame, 2—measuring cell, 3—lid of shear cell, 4—tie rods, 5—counterweight, 6—suspension rod, 7—strain gauges, 8—lever load system, 9—moving weights, 10—main switch, 11—PC with evaluation software.

The angle of internal friction of all samples tested was determined using the Schulze RST-01 rotating ring shear tester (Fig. 3).

The samples (P1–P5, R1–R5, CP and P MIX, R MIX) are inserted into the measuring cell (2). The surface of the inserted material in the cell is aligned according to the upper surface of the cell and weighed, to determine the volume of material. Next, the cell is inserted into a rotating base, which transmits the torque of propulsion to the measuring cell (2). The drive is located in the machine frame (1). Against the rotation of the sample-filled cell, the lid blades (3), which are embedded in the material, act through the loose material. The rotating drift of the lid (3) is prevented by the tie rods (4), which are connected to the strain gauges (7) and sense the pulling force generated by the rotation of the cell (2). For measurements, the lid (3) is loaded with normal stress through a suspension rod (6), which is connected via a strain gauge to a lever load system (8), along which moving weights (9) adjust the size of normal force. A counterweight (5) always returns the lid to the starting position. The measurement results in a shearing between the material drifting with the lid blades (3) and the material drifting by the cell (2). It creates a shear area in which the particles move and resist each other (shear stress). This shear stress, depending on the adjusted normal load, is then converted using software (Schulze—RST CONTROL 95) (11) to the internal friction efficient (AIFE), linearised (AIFLIN) and steady-state flow (AIFSF)^33^. Each sample was measured 10 times at a normal load of 20,000 Pa, 10 times at 10,000 Pa and 10 times at 5000 Pa.

#### Static and dynamic angle of repose

**Figure 4:**
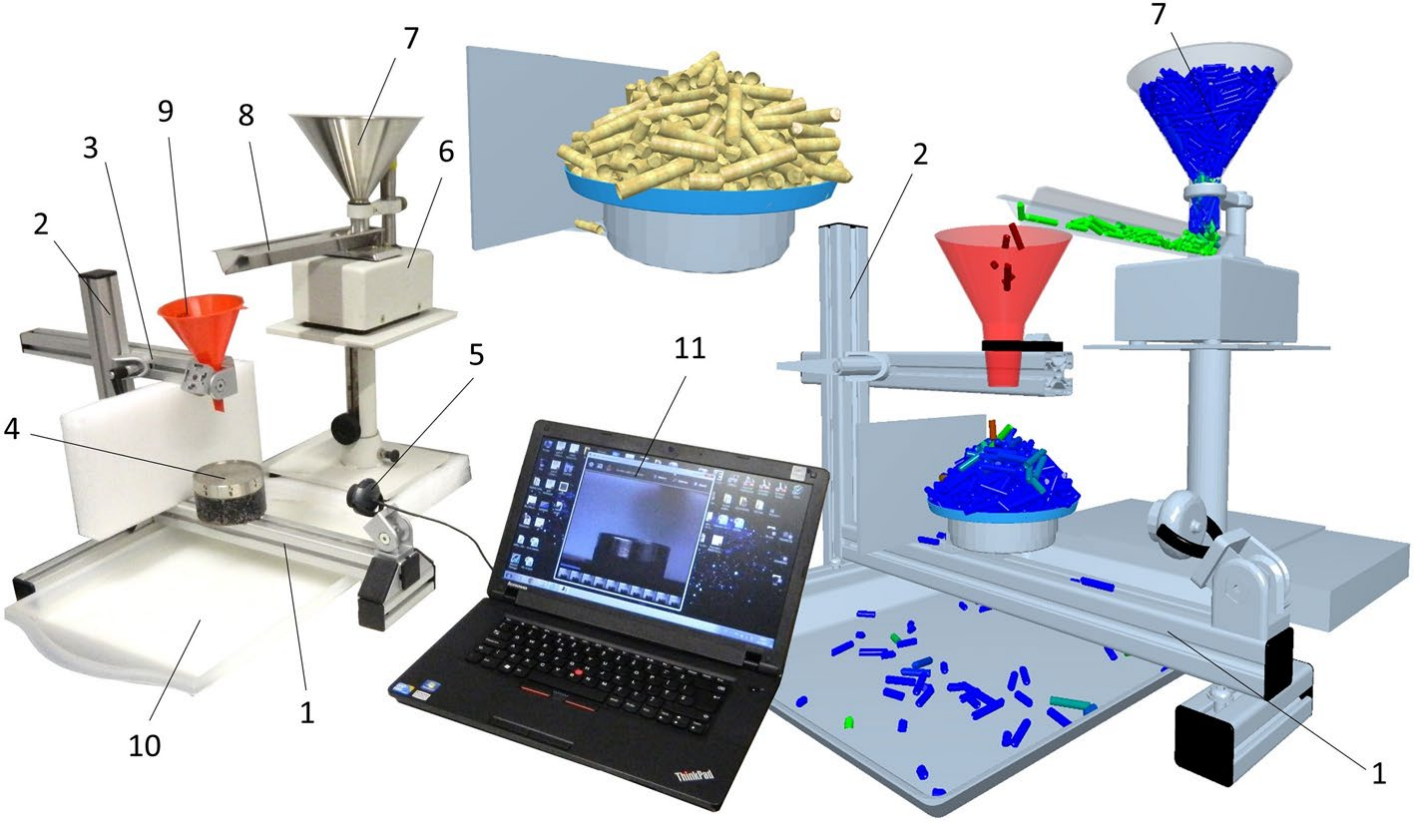
Schematic representation of Zenegero device to determine static angle of repose 1—device frame, 2—column profile to adjust shoulder height, 3—shoulder, 4—circular cell, 5—camera, 6—vibrating feeder, 7—filling funnel, 8—transport trough, 9—routing funnel, 10—collecting tray for excess material, 11—PC with software.

The static angle of repose of all samples tested was determined using a proprietary device from the patent PV2015-239 Validation device and method of measuring static and dynamic angle of discharge VSB—Technical University of Ostrava^34^. The device, the so-called Zenegero, is shown schematically in Fig. 4.

The samples tested are vibrated through a vibrating feeder and then through a conical hopper, and gradually brought onto a stainless-steel rotating dish. The material on the rotating dish is continuously weighed to determine when there is no further weight gain (another material is poured outside the stainless-steel rotating dish down the slope onto the mat). The camera takes images of the slope from 8 different sides. Graphic post-processing evaluated the average angle of repose.

**Figure 5:**
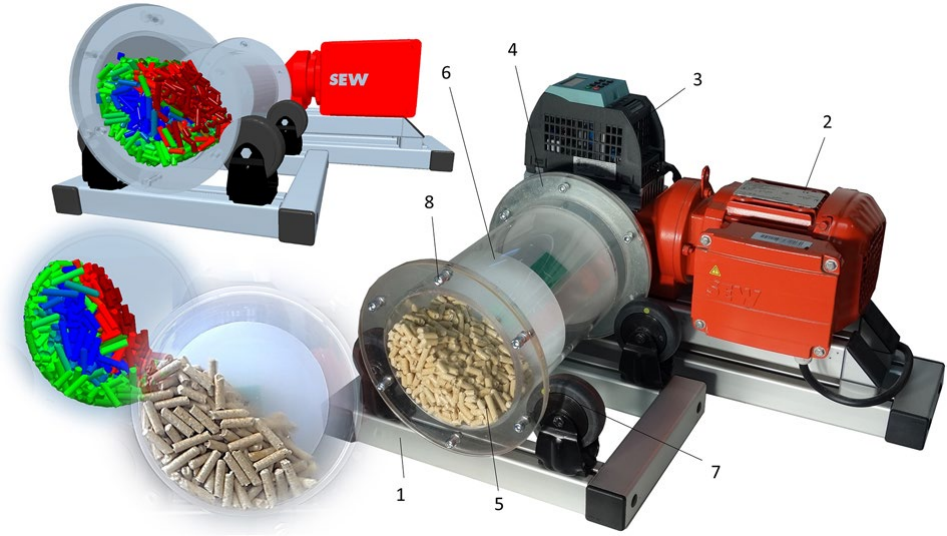
Schematic representation of a device for determining a dynamic angle of repose: 1—main frame, 2—electric gearbox, 3—frequency converter, 4—transparent drum, 5—transparent drum lid, 6—circular partition to reduce the volume of the drum, 7—pulleys, 8—screw connection.

The dynamic angle of repose (DAOR) of the test samples was measured in a device (Fig. 5) consisting of a main frame (1) on which the electric gearbox (2) is mounted, the output of which is firmly connected to a rotating transparent drum (4) rotating on pulleys (7)^35^. In this rotating drum (4) with a diameter of 0.140 m and a width of 0.03 m (Fig. 5), a circular partition (6) is inserted to reduce the volume of the space filled in the testing with pellet samples (P1–P5, PMIX), rods (R1–R5, RMIX) and CP crushed pellets. The drum was closed with a transparent lid (5). The fill rate for each measurement was 45%. The rotation frequency was set using the frequency converter (3) at 0.2, 0.4 and 0.6 Hz. The dynamic angle of repose was evaluated for all samples for each set frequency 10 times.

### Validation vibrating conveyor, experimental setup

**Figure 6:**
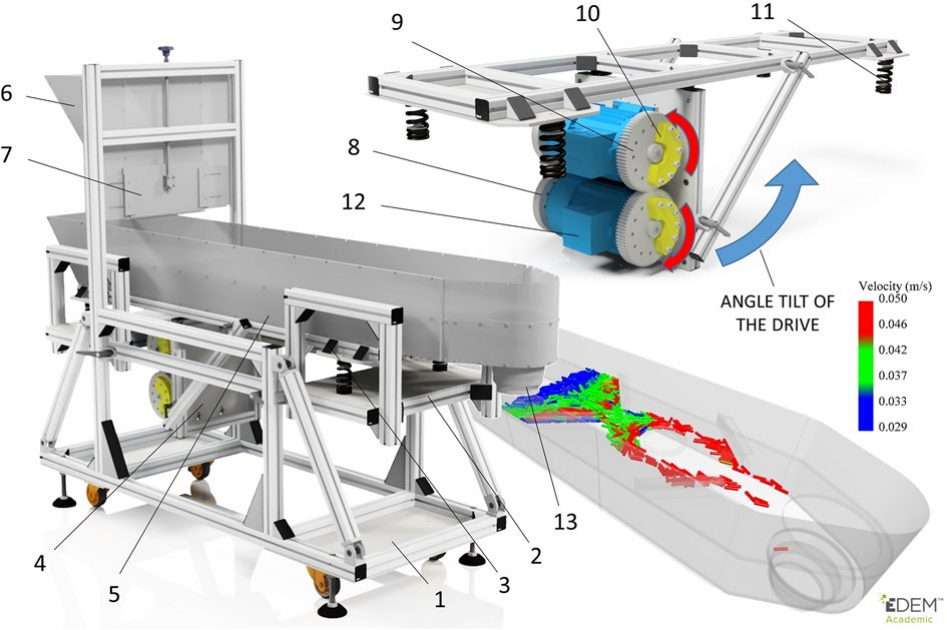
Description of the vibrating conveyor: 1—main frame, 2—middle frame, 3—upper frame, 4—tilting frame, 5—transparent transport trough, 6—hopper, 7—opening flap, 8—supporting wheel, 9—gear wheel, 10—weights, 11—springs, 12—electric engine, 13—out-hopper.

**Figure 7:**
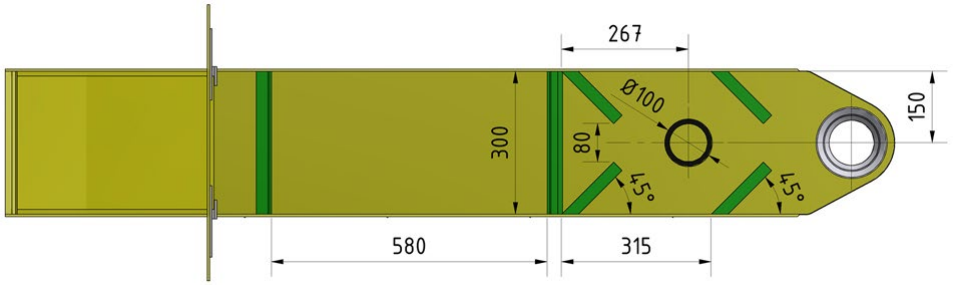
Dimensional drawing of the deployment of passive elements on the vibrating trough—view from above.

The vibratory behaviour of the pellets was investigated on a validation vibrating conveyor (Fig. 6). This same device is also used to compare the simulation calculations in the Discrete Element Method (DEM) with reality. It consists of a main frame (1) on which a middle frame (2) is placed, and an upper vibration frame (3) is attached by springs (11). Two electric motors (12) are placed on the vibration frame, which have supporting and gear wheels (8, 9) with weights (10) at the ends, and vibrations are created due to the imbalance of these wheels. With the rate of rotation of the imbalance weights and the tilt of the drives that are mounted on the tilting frame (4), we adjust the amplitude and frequency of the vibrations of the transport trough (5). In this case, the rotation frequencies were set at 15, 17.5, 20 and 22.5 Hz and the tilt of the drives was set to 40 degrees relative to the trough (5). The transport speed and behaviour of each sample (P1–P5, P mix, CP, R1–R5, R MIX) on the length of the 580 mm transport line were first observed. Measurements were taken with a volume of 800 ml, which was always placed at the back of the trough (5). Each sample was measured 10 times. Thereafter, pellet behaviour was observed and validated with an equal volume of each sample (800 ml) for flow through passive elements, see Fig. 7.

### Sensing technique

The vibration size of the validation vibrating feeder (Fig. 6) was scanned with a high-speed camera (Lavision—Imager HS 4lM) and the data was evaluated using i-Speed Software Suite tracer software. The validation device sensed from three directions where desired vibrations in the direction of movement of the sample and undesirable side vibrations of the machine were detected. Vibrations in the direction of movement of the sample were taken at three points, from the hopper (6), above the electric engine (12) and at the out-hopper (13). Side vibrations were taken from the front of the hopper (6) and the front of the out-hopper (13) (Fig. 6). The sensed distances at each location, for selected frequencies, are shown in Table 2.

**Table 2:** Vibration size of the x and y validation vibrating feeder.

Vibration frequency (Hz)	Vibration in direction of motion						Side vibration			
	Hopper		Engine		Out-hopper		Hopper		Out-hopper	
	x (mm)	y (mm)	x (mm)	y (mm)	x (mm)	y (mm)	x (mm)	y (mm)	x (mm)	y (mm)
15	0.90	1.23	0.77	1.34	0.90	1.60	0.11	1.11	0.14	1.78
17.5	0.87	1.21	0.79	1.38	0.84	1.53	0.12	1.24	0.16	1.62
20	0.77	1.39	0.81	1.61	0.80	1.64	0.21	1.48	0.16	1.64
22.5	0.76	1.71	0.84	1.86	0.72	1.65	0.06	1.83	0.14	1.82

**Figure 8:**
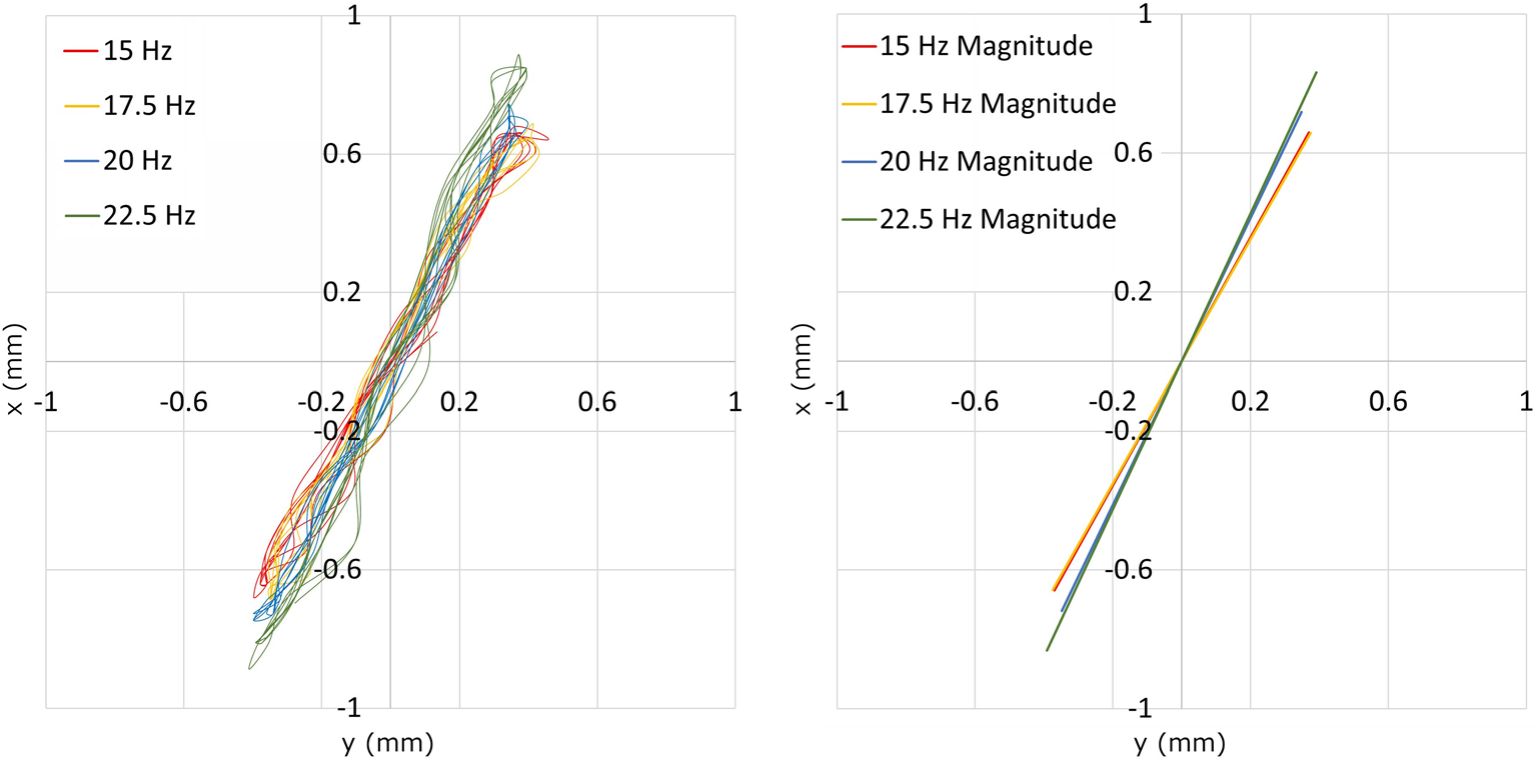
The direction of vibrations (left) and magnitude vibrations (right) of the selected vibration frequencies.

For the subsequent use of the data, vibrations above the engine were chosen (Table 2), since above this area there is also a measuring area of transport. Based on the evaluation of the data from the trace program, a graph has been created showing the course of vibrations of the transport trough for the selected frequencies (15, 17.5, 20 and 22.5 Hz) in the time span of 0.3 s (Fig. 8 left). By linearising the raw data, magnitude vibrations were created for the selected frequencies (Fig. 8 right).

## Results

### Particle size distribution and particle shape

As part of the material characterisation, the particle size distribution of the individual samples (Table 3) was determined to compare the determined values with other mechanical-physical parameters of the tested materials.

**Table 3:** Characteristics values for the particle size distribution.

	d10 (µm)	d50 (µm)	d90 (µm)	Span S (–)
P1	6194	6479	6808	0.09
P2	8027	9502	10,459	0.26
P3	9695	11,287	12,208	0.22
P4	11,570	12,971	13,709	0.16
P5	11,936	14,113	14,890	0.21
P MIX	8754	11,666	14,122	0.46
CP	68	375	2551	6.62
R1	6778	7327	7754	0.13
R2	8907	9475	9819	0.10
R3	11,058	11,470	11,793	0.06
R4	12,778	13,287	13,639	0.06
R5	14,058	17,733	15,077	0.07
R MIX	8808	11,400	14,207	0.47

**Figure 9:**
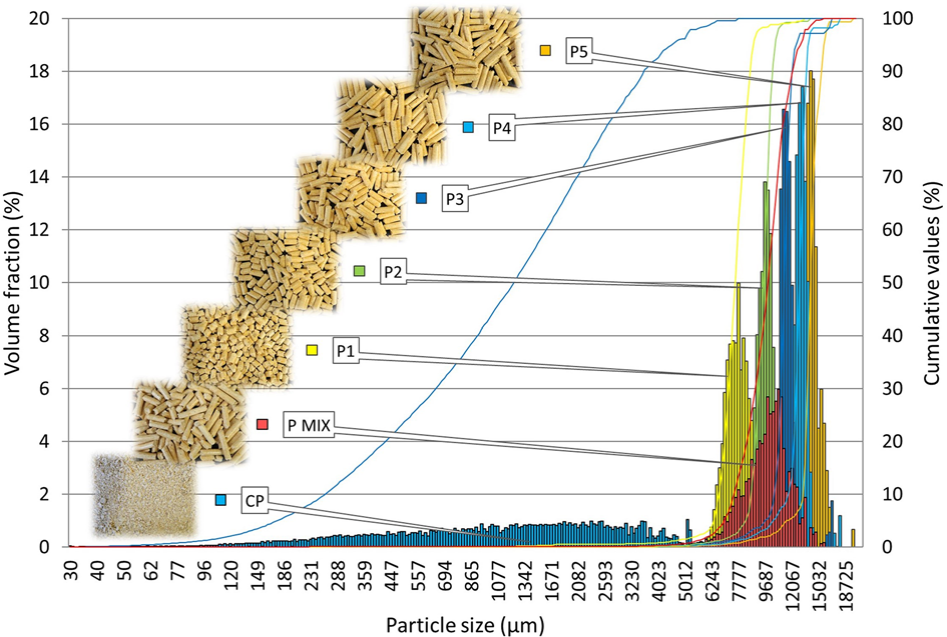
The particle size distribution for pellets.

**Figure 10:**
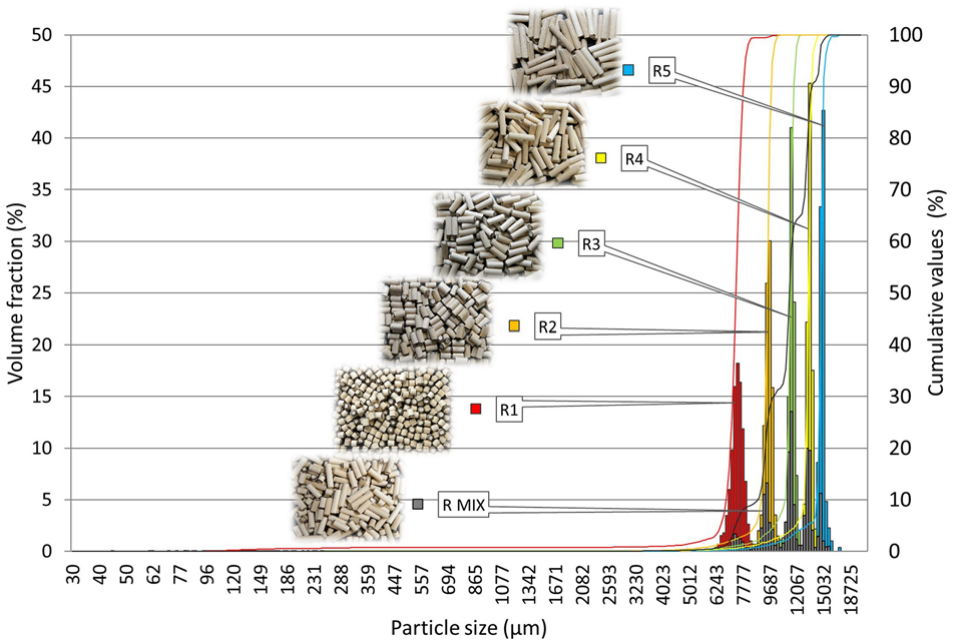
The particle size distribution for rods.

For the distribution of spruce pellets (Fig. 9), it is possible to observe the distribution of individual samples, which were formed by splitting individual length representatives from the P MIX sample (the distribution is shown in red). This distribution serves, in principle, as a control and shows the percentage of individual length representatives. The reverse procedure was applied to the sample from the wooden rods where the RMIX mixture was prepared (mixed) according to the measured PMIX sample distribution, which is seen in Fig. 10. The percentage of length representatives is the same, though the sample distribution is not only unimodal, but a five-modal distribution can logically be observed, as the RMIX sample does not, like PMIX, also contain other length representatives of pellets. When comparing the two distributions, the spruce pellet samples appear to be in greater dispersion, indicating that the samples are not of equivalent length and ideal shape. The significant distribution difference between PMIX and CP crush is apparent, suggesting different behaviours between the two samples.

**Figure 11:**
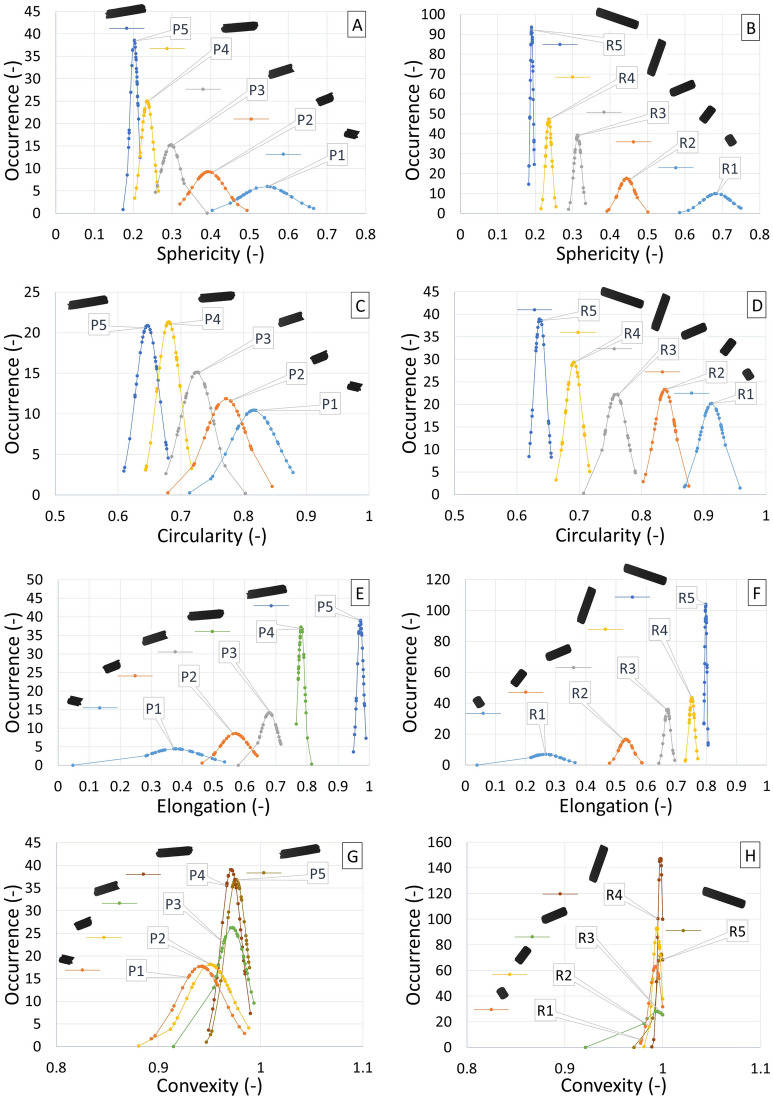
Particle shape—sphericity, circularity, elongation, convexity of sorted pellets and rods.

Non-scale-dependent parameters have been purposefully selected to determine the shape of particles for all samples. All particle shape parameters are dimensionless (Table 1). These are sphericity, circularities, elongation, convexity. Their measured values are given in Table 4 and Fig. 11, respectively. From the given data, it is possible to easily describe not only the shape of the particles tested, but also to estimate the relative structure and relative size of the surface. The graphs for sphericity, circularity and elongation parameters in Fig. 11 show the representation of the major range of measured parameters, i.e., the occurrence on the x-axis. In the case of convexity, this representation is logically narrower. The sphericity and circularity data for P1–P5 and R1–R5 samples decreases with the increasing elongation parameter (Table 4). It is therefore clear that the length of the particle can also be estimated from the sphericity and circularity values. With SPH and CIR close to 1, the particles are similar to a circle or a sphere (ideal bulk). The ELO (length) value of such particles is therefore lower. This linkage of the tested parameters is also apparent from the graphs (Fig. 11), where the order of the individual samples P1–P5 and R1–R5 is opposite to the Sphericity and Circularity for the Elongation parameter.

**Table 4:** Characteristics values for the particle shape.

	Sphericity		Circularity		Elongation		Convexity	
	SPH (–)	σ_sd_ (–)	CIR (–)	σ_sd_ (–)	ELO (–)	σ_sd_ (–)	CON (–)	σ_sd_ (–)
P1	0.54	0.07	0.82	0.04	0.38	0.09	0.94	0.02
P2	0.39	0.04	0.77	0.03	0.57	0.05	0.95	0.02
P3	0.29	0.03	0.73	0.03	0.68	0.03	0.97	0.02
P4	0.24	0.02	0.68	0.02	0.74	0.02	0.97	0.01
P5	0.20	0.01	0.65	0.02	0.78	0.01	0.98	0.01
P MIX	0.35	0.10	0.75	0.06	0.62	0.10	0.97	0.01
CP	0.45	0.22	0.87	0.08	0.46	0.17	0.97	0.07
R1	0.68	0.04	0.91	0.02	0.27	0.06	0.99	0.01
R2	0.44	0.02	0.84	0.02	0.53	0.02	0.99	0.01
R3	0.31	0.01	0.76	0.02	0.67	0.01	0.99	0.01
R4	0.28	0.01	0.69	0.01	0.75	0.01	0.99	0.01
R5	0.19	0.01	0.63	0.01	0.80	0.01	0.99	0.01
R MIX	0.36	0.02	0.77	0.02	0.63	0.02	0.99	0.01

When comparing all the shape parameters of the P1–P5 pellet and R1–R5 rods, a greater variance is always seen in the case of P1–P2 pellets. The samples are not of equivalent length and ideal shape. These conclusions are also consistent with particle size distributions (Figs. 9, 10).

An interesting output from the measured P1–P5 sample shape parameters is the possibility to predict the representation of individual size fractions in the PMIX sample. This example is shown in blue in Table 4. The SPH 0.35 and CIR 0.75 values for P MIX predict quite obviously a major representation of P2 and P3 particles. In percentage terms, this conclusion is also confirmed by the measured particle size distribution.

The next parameter evaluated was Convexity. It is commonly known that the smoother the surface (no protrusions, edges, thorns), the closer the CON value is to 1. The P1–P5 spruce pellets showed CON values in a range of 0.94–0.98. This confirms that these pellets are not ideally smooth, and their ends have burrs. The opposite is the case for R1–R5 rods, where the results of CON (0.99) show a uniform smooth surface, the same for all samples.

For the R MIX sample, the above-mentioned prediction of representation from the data already measured was used. The R MIX sample was artificially created by mixing the proportional representation of the R1–R5 size fractions according to the P MIX model to maintain the consistency of spruce pellet and rod samples. The shape parameters were therefore calculated for the R MIX sample according to the percentage of R1–R5 particles (R1—10%, R2—24.4%, R3—34%, R4—22.6%, R5—8.9%) for which all parameters had already been evaluated. From the calculated values it is clear that R MIX is similar to the P MIX sample (Table 4, marked in red).

The mixture thus created is particularly useful because of the calculations in the DEM method (“Effect of vibrating conveyor setting on particle transport speed” section). An appropriate compromise to determine the number of rod length representatives is to set the complexity of the simulation and therefore also the time of the simulation calculation^36^. Shape is also an important parameter regarding the formation of the particle itself in the DEM method and thus complements the description of loose matter.

### Angle of internal friction

**Figure 12:**
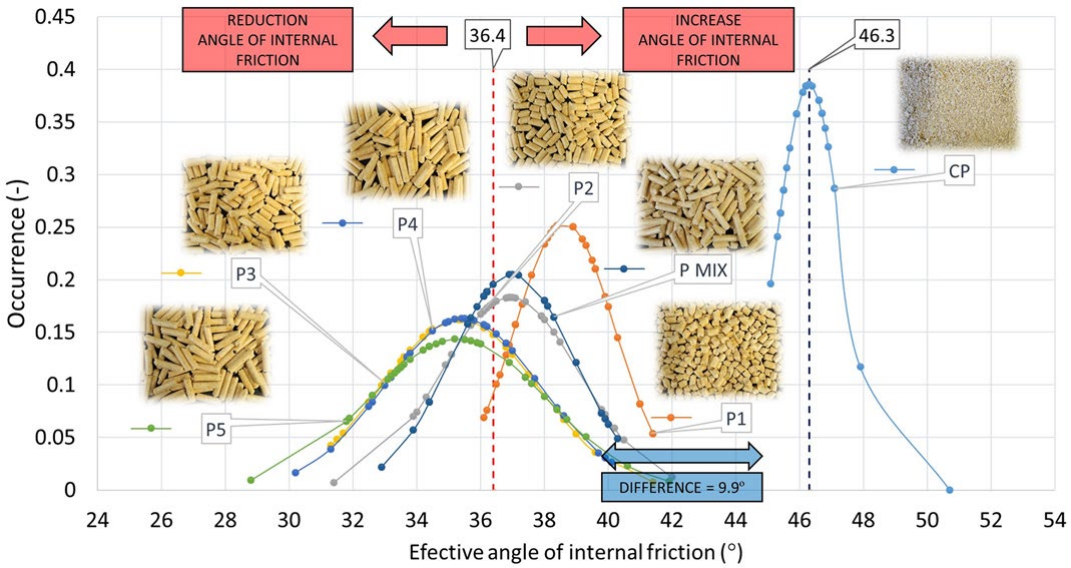
Effective angle of internal friction pellets P1–P5, PMIX and CP.

**Figure 13:**
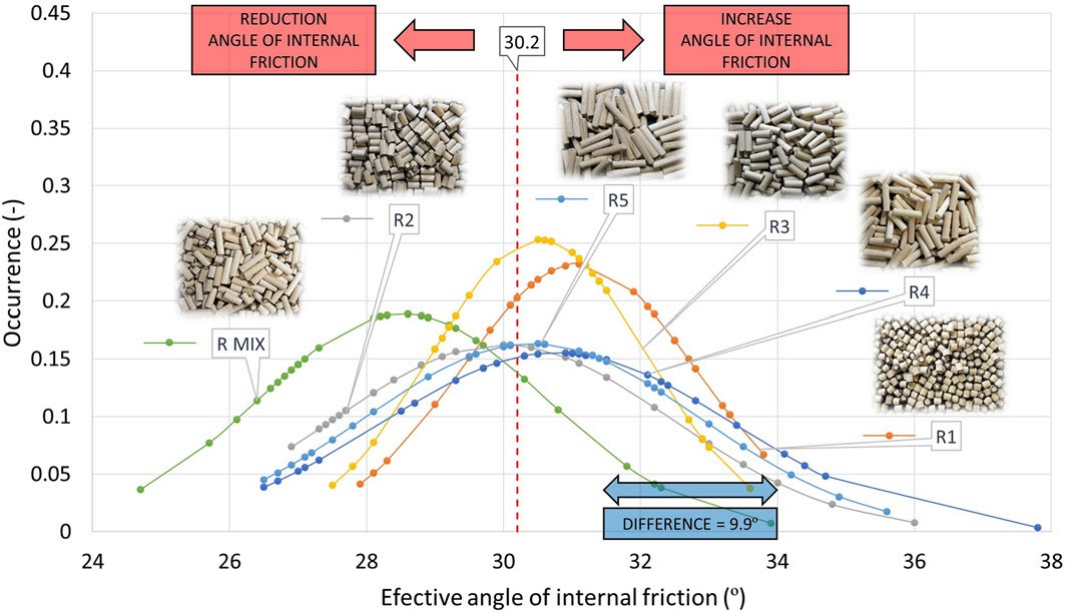
Effective angle of internal friction pellets R1–R5 and RMIX.

The evaluation of all specified angles of internal friction, both effective (AIFE), linearised (AIFLIN) and steady-state flow (AIFSF) is presented in Table 5, Figs. 12 and 13, respectively.

**Table 5:** Internal friction (AIFE, AIFLIN and AIFSF) with standard deviation.

	AIFE (°)	σ_sd_ (°)	AIFLIN (°)	σ_sd_ (°)	AIFSF (°)	σ_sd_ (°)
P1	38.6	1.6	38.3	1.5	36.1	1.0
P2	36.9	1.6	36.7	2.3	35.3	1.6
P3	35.3	2.5	35.2	2.4	34.1	1.7
P4	35.4	2.5	35.2	2.4	34.1	1.9
P5	35.3	2.8	35.1	2.7	34.2	2.7
P MIX	37.0	1.9	36.8	1.9	35.2	1.6
CP	46.3	1.0	43.6	0.7	42.4	0.3
R1	31.1	1.7	30.9	1.7	29.5	1.5
R2	30.0	2.5	29.8	2.5	29.4	2.3
R3	30.5	1.6	30.4	1.5	29.7	1.4
R4	30.8	2.6	30.7	2.5	30.2	2.3
R5	30.4	2.4	30.2	2.3	29.9	2.2
R MIX	28.5	2.1	28.4	2.1	27.7	1.8

For the mutual comparison of the samples, AIFE (Table 5) has been selected from the overall measurement of internal friction angles, which shows the highest values and therefore provides the greatest safety with regard to the design given the functionality of the device and processes. The work of Dafnomilis gives higher values of effective internal friction angles for wood pellets^37^. In the range of 39°–45°. However, this may be due to their different mechanical resistance.

As expected, the CP sample was found to have a different friction than the P1–P5 sample group, as well as PMIX, as shown in Fig. 12. In this case, friction is 9.9° higher than the average value of the effective angle of internal friction of the P1–P5 samples. In terms of comparing the angles of internal friction to the shape of the particles, one would assume that the more the shape of the pellet is closer to the sphere, the less friction will occur.

However, for oblong particles, it must be taken into account that these particles are arranged in the direction in which the cell rotates (in the direction of movement) during the measurement, hence their friction is reduced (according to the shape of the sphere). The arrangement of particles in the cell is not rectangular, but circular. Therefore, longer P2–P5 particles show less friction than P1 particles. When comparing the internal friction angle of the P1–P5 and PMIX pellets to the R1–R5 and RMIX rods, it can be observed, with respect to the solidity of the R1–R5 and RMIX particles (no degradation during measurement), and further depending on the shape of the termination of the R1–R5 and RMIX particles (straight without burrs), that the mean of R1–R5 and RMIX is 6.2° lower than P1–P5 and PMIX. It follows that the production of pellets alone, due to their more precise shape and higher strength, can ensure a reduction in friction, and hence a reduction in energy for the transport of the particles thus produced. The pellets shape therefore plays a dominant role and obvious effect on the flow characteristics. Similar results, but in the context of pneumatic conveying, were obtained in a study by Liu^38^, where particles of different length-to-diameter ratios were compared. Particles in the ratio equal to one always behaved differently from other particles. Similarly, in our case, the P1 (length/diameter ~ 1) values are also noticeably different in the measured properties.

Furthermore, it can be observed that the degradation of pellets may act as a lubricant in small concentrations. However, if the sample degrades for the most part, or completely, the transport costs of this mixture will in turn increase, as shown by the mean AIFE of CP = 46.3°.

This conclusion can be supported by the classification of samples into individual flow modes (Table 6), where the sample CP is included in the Cohesive group, confirming the increased transport problems of this sample.

**Table 6:** Static AOR with standard deviation of measurement (σ_sd_) and classification of flow properties.

	AOR (°)	σ_sd_ (°)	Flow properties
P1	37.7	1.8	Free flowing
P2	38.0	2.4	Free flowing
P3	37.7	2.6	Free flowing
P4	35.4	2.7	Free flowing
P5	35.5	2.4	Free flowing
P MIX	36.7	2.2	Free flowing
CP	47.0	2.0	Cohesive
R1	32.9	1.5	Free flowing
R2	36.6	2.2	Free flowing
R3	33.2	2.7	Free flowing
R4	33.1	2.2	Free flowing
R5	30.6	3.5	Free flowing
R MIX	33.7	2.2	Free flowing

### Static and dynamic angle of repose

Table 6 provides the static angle of repose (AOR) values together with classification into individual flow modes^39^.

**Figure 14:**
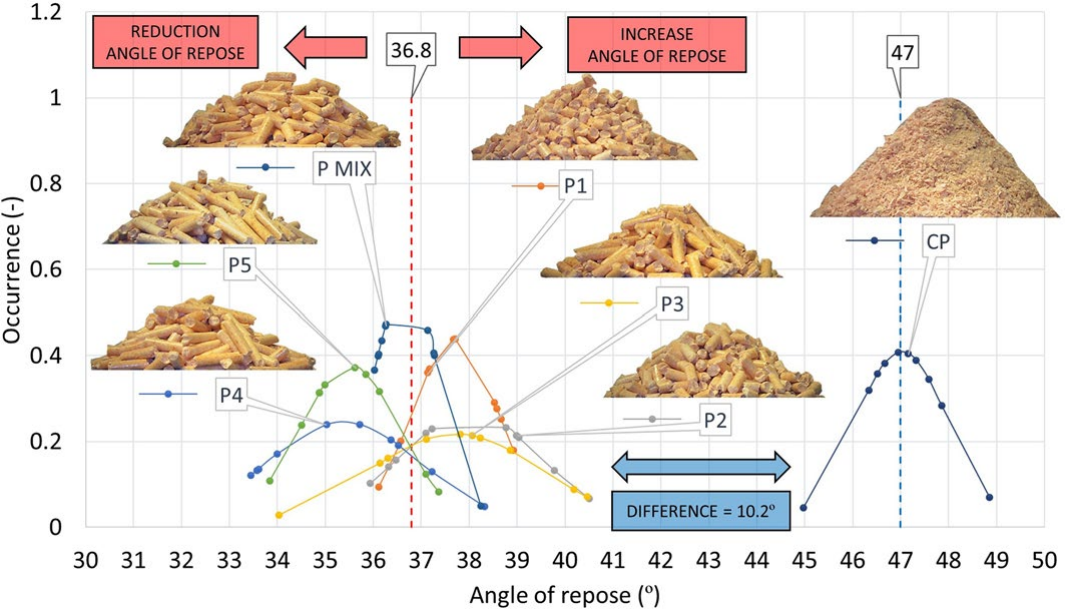
Angle of repose pellets P1–P5, PMIX and CP.

The table shows that all samples, except the CP sample, fall under the Free flowing mode of the evaluation methodology^40^. This flow mode is suitable for the transport and handling of samples. Smooth processing with respect to sample flow can be predicted. These results are in relative agreement compared to the literature data^41^. It is clear from the results that the average is made up of higher values of shorter pellets (P1–P3) and lower values of longer pellets (P4–P5). The CP sample falls into the Cohesive category. It can, therefore, cause problems in transport. All values of the static repose angles of the rods are lower compared to the pellets. The measurement of the static angle of repose confirms the results arising from the determination of the effective angle of internal friction. Also related to this is the fact that the average AOR of the CP sample shows a similar difference in this case from the average AOR of the P1–P5 and PMIX samples, of 10.2° (Fig. 14), identical to the values of the effective angle of internal friction (Fig. 12).

**Figure 15:**
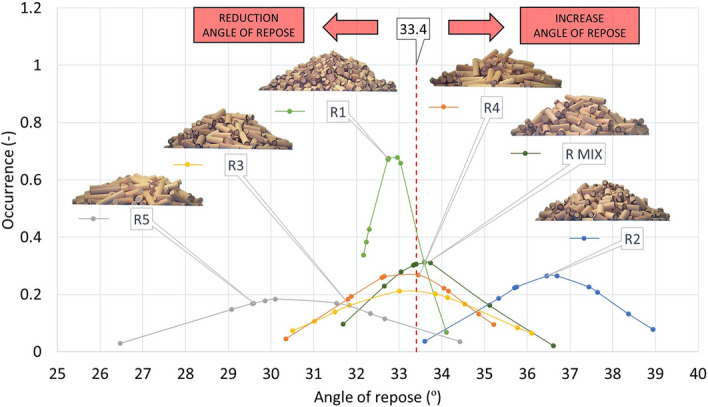
Angle of repose rods R1–R5 and RMIX.

From the results of the measurements of the angle of repose pellets (P), crushed pellets (CP) and rods (R) (Figs. 14, 15), it can be concluded that these are similar results to those of the measurements of the effective angle of internal friction. It is also evident that the mean value of the angle of repose rods (R) has decreased by 3.4° from the mean value of the angle of repose pellets (P). This decrease is due to the shape of the measured particles, where the rods (R) have a more precise particle shape with aligned end surfaces, higher strength, and therefore, a lower average angle of repose value.

An interesting observation, compared to the results of the effective angle of internal friction and the angle of repose, is the difference in achieving the highest values. These are the shortest: P1 and R1 particles for AIFE and P2, respectively, and R2 for AOR. This difference is due to the way the pellets are arranged in the formation of the pile, where the sample particles (P2, R2) are not fully able to be arranged in the path of least resistance, as they are not affected by a significant normal force (set value), as in the AIFE measurement, where 20, 10 and 5 kPa (“Angle of internal friction” section) have been set sequentially. Through this application of normal force and rotation over an infinite circular path, the sample particles are turned in their shape to offer the least resistance to each other in the shear volume.

**Figure 16:**
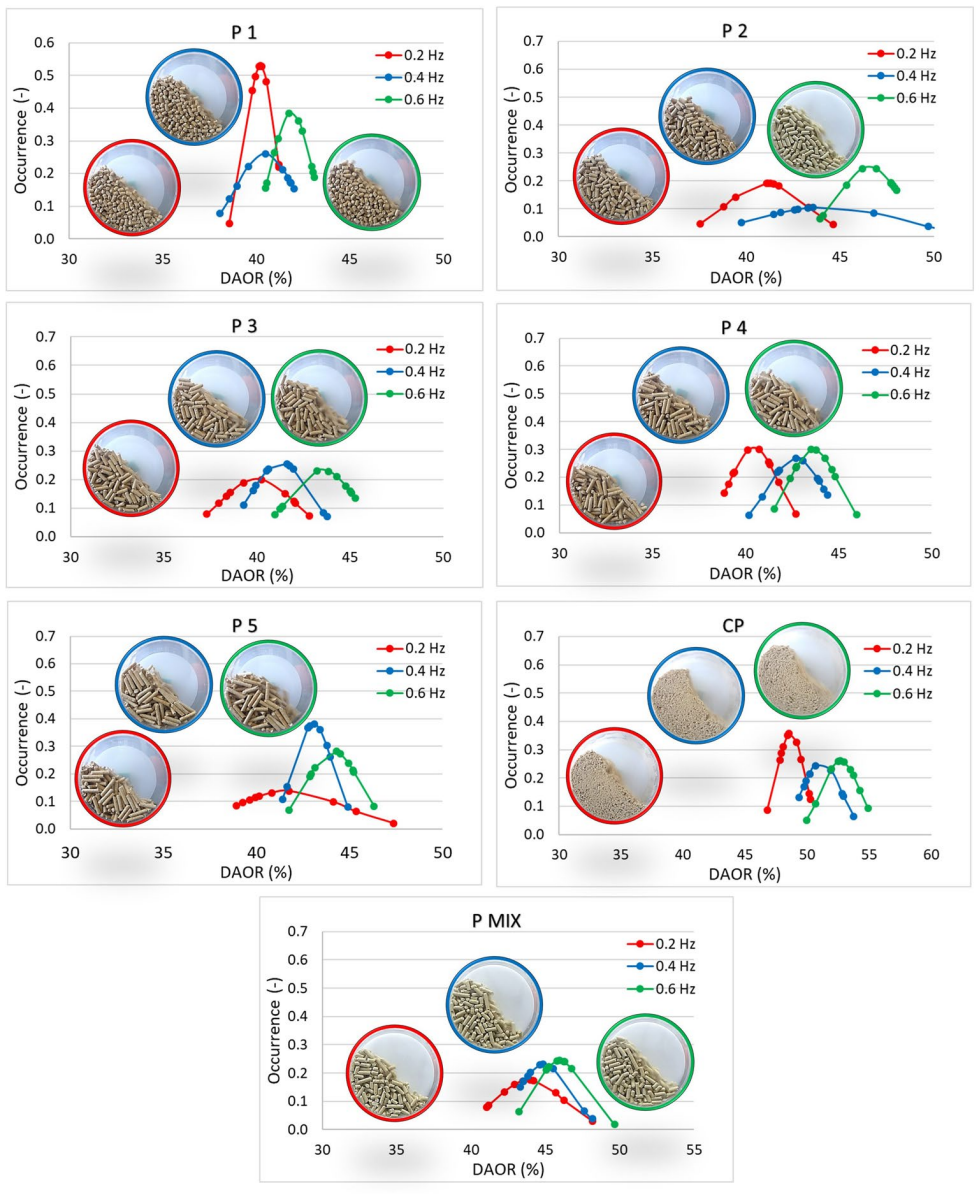
Dynamic angle of repose (DAOR) for pellets and CP.

**Figure 17:**
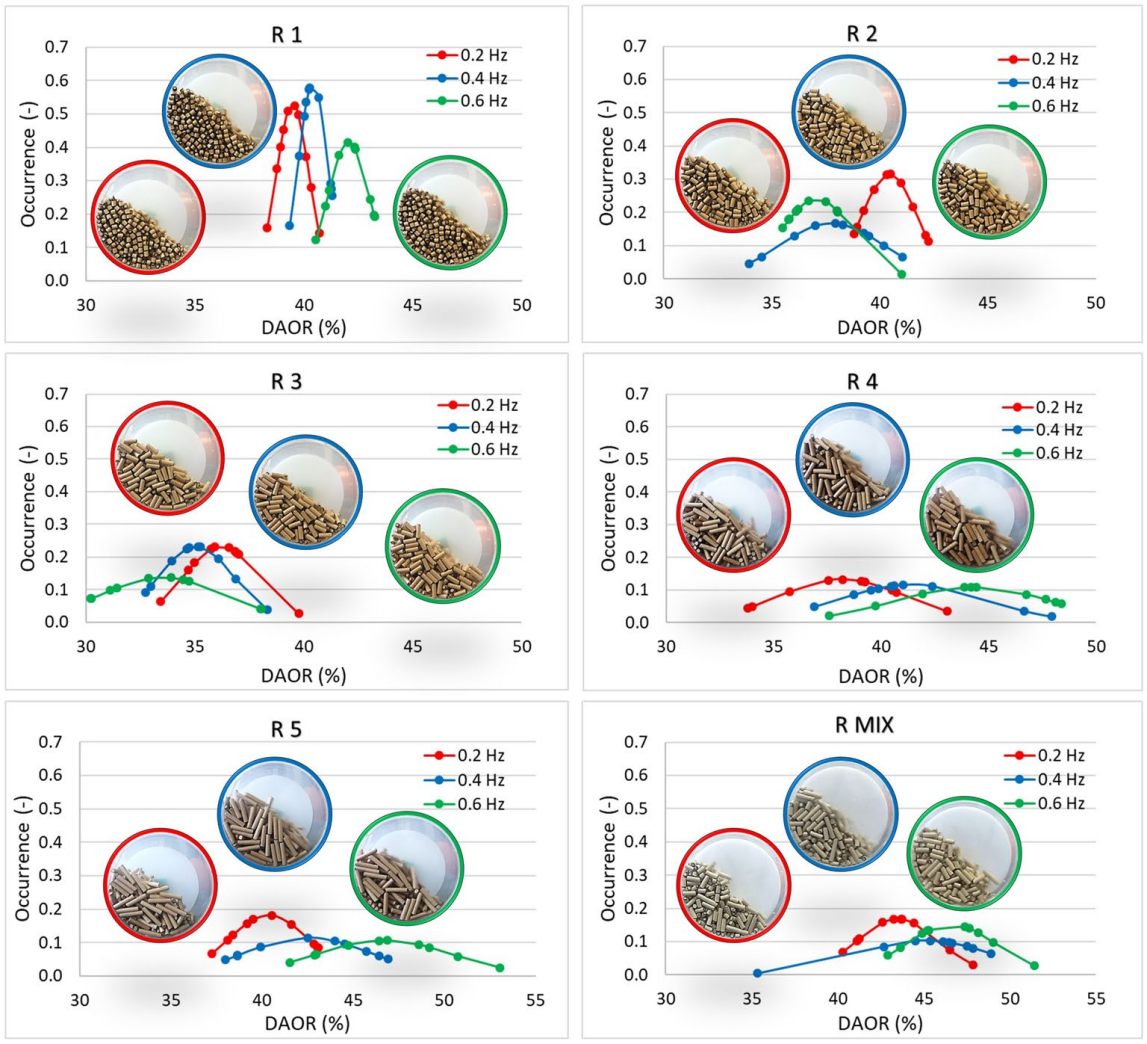
Dynamic angle of repose (DAOR) for rods.

Figures 16 and 17 plot dynamic angles of repose values for frequencies of 0.2, 0.4 and 0.6 Hz for pellet (P) and CP samples and for rods (R) respectively. The stability of the dynamic flow with increasing frequency for individual samples was assessed in these measurements, but the samples were also compared to each other overall.

The strongest flow with increasing frequency is evident for sample P1 (Fig. 16). There is no significant change in the size of the angles read; in all cases, there was a cascading movement of particles in the drum. A significant change can be observed in the case of CP. DAOR values for CP are highest (53° for 0.6 Hz). By behaving, the CP sample can shift at a frequency of 0.4 and 0.6 Hz to the cataracting mode.

For rod samples (R), R1 (Fig. 17) is the most stable with the increasing frequency of the rotating drum. In this case, a rolling flow mode is seen, which also continues for the R2 and R3 sample. In the case of R5, long rods were blocked during mixing, due to the random rotation of the particles in different directions. The induced blocking of these individual particles added to the dynamic angle of repose at different intervals, making it difficult for other particles to pass through. The mixing intensity (kinetics) in this case would be very low. The DAOR values of sample R5 reach up to 53° at 0.6 Hz.

Again, similarities can be observed with the AIFE and AOR results, where the DAOR value for rods (R) is also lower in almost all cases than for pellet (P) samples (Figs. 16, 17). However, given the dynamics of the rolling motion process, the sliding of particles over each other in the rotary drum is the reason why, as with the AOR parameter, the longer the particles, the lower their AOR.

It appears that shape is one of the dominant parameters determining the arrangement of particles in a rotating drum. A cohesive CP sample, which can result from pellet degradation, is also undesirable from a DAOR perspective.

### Effect of vibrating conveyor setting on particle transport speed

**Figure 18:**
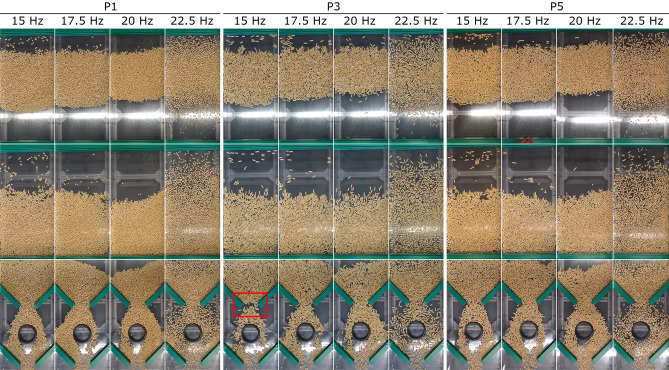
Pattern formation in real-time measurements of pellet transport speed depending on vibration frequency and pellet length.

**Figure 19:**
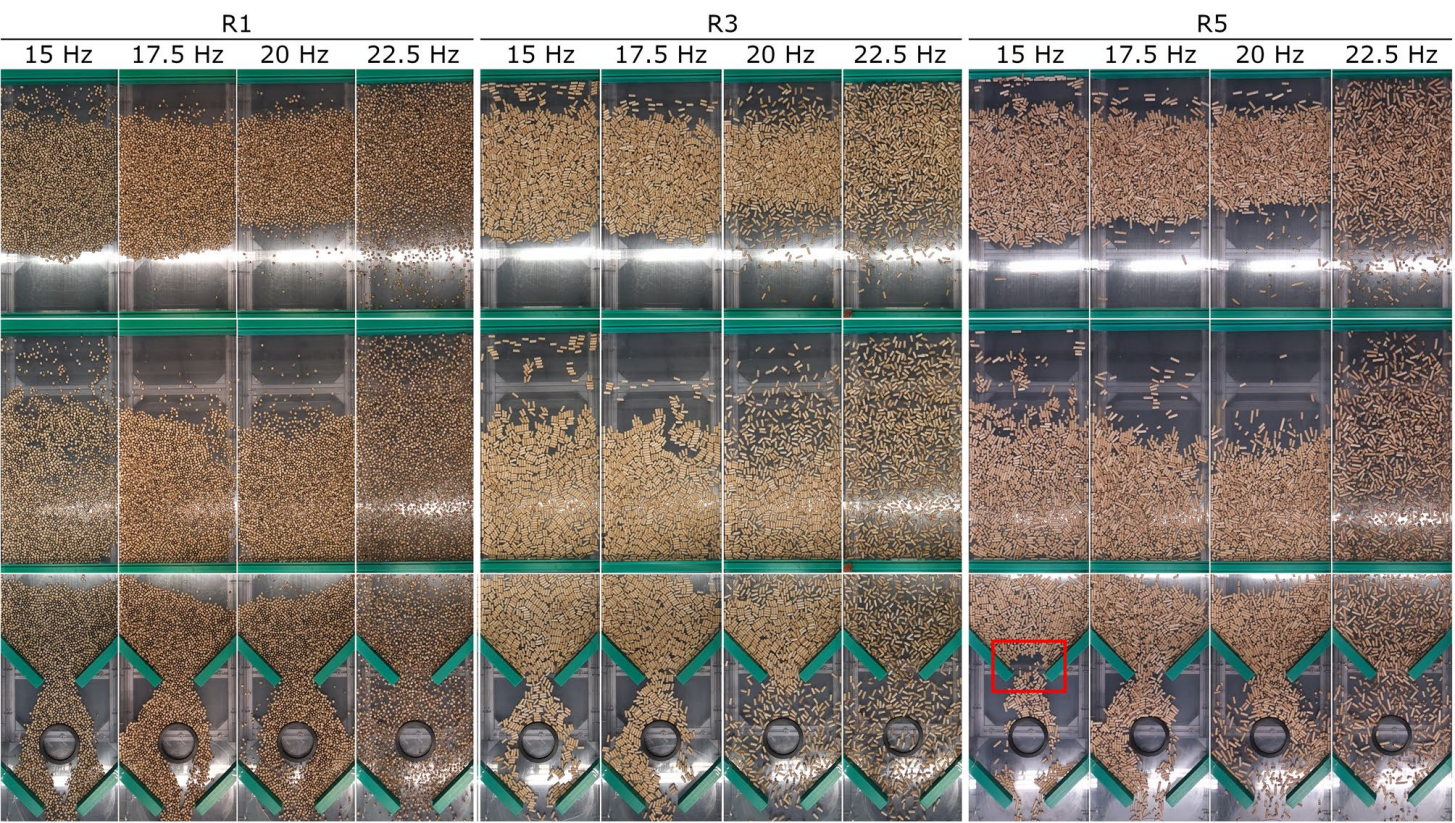
Pattern formation in real-time measurements of the transport speed of wooden rods, depending on the frequency of vibrations and the length of the rods.

To analyse, in detail, the vibration transport of the samples (R and P) and to compare real measurements of the transport speed of pellets and wood rods, the vibrating conveyor was scanned during individual measurements. Photographs were taken of the selected videos showing the dominant points during the real measurement for selected sample sizes P1, P3 and P5 (Fig. 18) as well as R1, R3 and R5 (Fig. 19). The images always show the flow of material in the stretch of vibrating conveyor in which the speed of the traffic was measured (Figs. 18, 19 series 1 and 2) and in the passive segment (Figs. 18, 19 series 3).

The real measurements of pellets P1, P3 and P5 (Fig. 18) indicate structure formation. The first sign of the structures appears in the central area of the vibrating trough. This is in good agreement with the findings of references^1,6,42^. At a low frequency of 15 Hz, the material tends to form stripes (a mass) of particles, a so-called stripe formation, which shorten (thinner) at higher frequencies. This rule ceases to apply at a frequency of 22.5 Hz, and pellets tend to move in the stripe (mass), but the high frequency causes part of the pellets to move faster and part of them to move more slowly. This disperses the pellets over the entire area of the sensed conveyor section. The stripes are unstable with this setting. On a stretch of conveyor with passive elements (Fig. 18, 3rd series), the material is first affected by a section with a reduced cross section of transport and then the flow is limited by a passive element in the form of a circular ring which the material must flow around. The third passive element is again the section with a reduced transport cross-section. In the first section with a reduced cross-section, when the section is evenly covered with material, there is a clogging of flow mass at low frequencies (predominantly 15 and 17.5 Hz) and larger pellet lengths (P3 and above). This is where the so-called arch (highlighted in red Fig. 18) is formed, which is also found in gravitational reservoirs. Higher frequencies eliminate these problems. Furthermore, it can be read that at vibration frequencies of 15 and 22.5 Hz, the material flows through the passive element of the ring evenly. For frequencies of 17.5 and 20 Hz, the transported material moves unevenly and move to the left side for a frequency of 17.5 Hz and to the right side for a frequency of 20 Hz, respectively. This can also be seen when changing the lenght of the tested pellets. This shows that the pellets length during their processing plays an important role, similar to the study of Lehtikangas^43^ where a noticeable effect was observed.

It can be said that this flow property derives from the production property of the machine too, especially the stiffness of the springs. However, this flow property can be used for separation (sorting), and by changing the frequency of the vibration it is possible to determine the direction of the movement of the material or where it will exit from the vibrating conveyor. Changing the vibrating conveyor settings would thus reduce the number of handling steps during pellet processing and reduce the degree of pellet degradation and associated risks^37^.

The same behaviour as for pellets can also be seen in the transport of wooden rods (Fig. 19). As wooden rods have less density than pellets, and thus less weight, they are more affected by the vibratory movement of the conveyor. In the section of traffic speed measurement, the behaviour is similar to that of pellets. Stripes are created, but they are wider and more difficult to recognise or define. The rod particles are more dispersed over the transported area than pellets. In the passive element section, the material also spreads more over the area of the conveyor. But the basic behaviour remains similar to the pellets. At frequencies of 17.5 and 20 Hz, the rods move on the left or the right side of the conveyor, respectively. At low frequencies (15 Hz) and higher dimensions (R3 and above), an arch is formed (highlighted in red, Fig. 19). It is clear from the pattern formation comparison that the formation of stripes is due to friction. The interaction between two particles and, at the same time, between the particles and with the contact material of the vibrating conveyor will not allow the particle to roll down the contact material of the conveyor. This creates a formation of particle clusters, or a formation of stripes. In the case of pellets showing a higher angle of internal friction (AIFE ~ 36°), this formation occurs more strongly. For rods that have smooth ends without burrs, this formation into stripes occurs to a lesser extent, also due to lower internal (AIFE ~ 30°) and wall friction.

**Figure 20:**
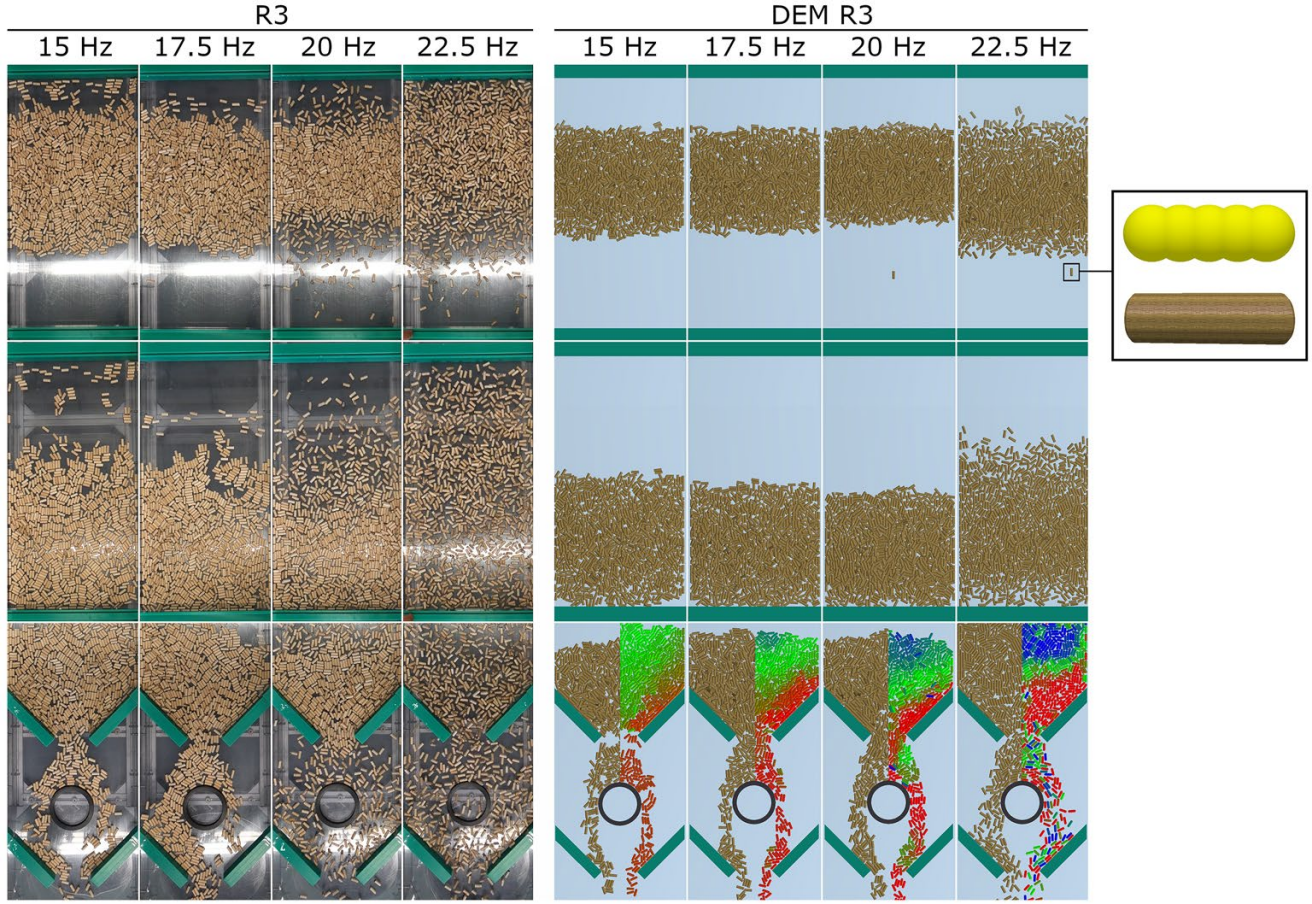
Comparison of real measurements and simulation calculations for R3 wooden rods.

The work also involved comparing real measurements and simulation calculations. The computer simulation was created using the EDEM Academic 2019 software. The simulation was based on the Hertz-Mindlin mathematical model (no slip)^36^. At the same time, a digital copy of the measurement device was created and the necessary restitution, static friction and rolling friction coefficients were determined to simulate movement^44^. The comparison used real measurement data from size R3 wooden rods and similar digital copies of the same diameter and length produced with 5 balls; for visualisation, this particle was interspersed with a template and texture reminiscent of the shape and appearance of a wooden rod (Fig. 20). The comparison of real measurements and simulation calculations (Fig. 20) suggests that digital simulations approach reality at low frequencies. A moving strip is created, and in part of the first narrowing of the passive elements, arches are formed. At higher frequencies, however, there is no dispersal of stripes over the larger area of the section being transported, and no dispersal of particles around passive elements. In order to achieve real particle behaviour in a DEM simulation for higher frequencies, the calibration of selected coefficients and particle properties is needed to achieve validation of the vibration transport process.

**Figure 21:**
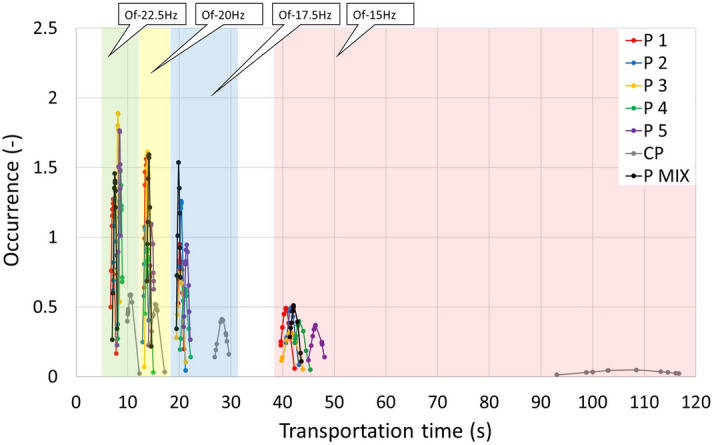
Transport time of particles when transporting pellets and crushed pellets, depending on the different frequencies of the transport distance.

**Figure 22:**
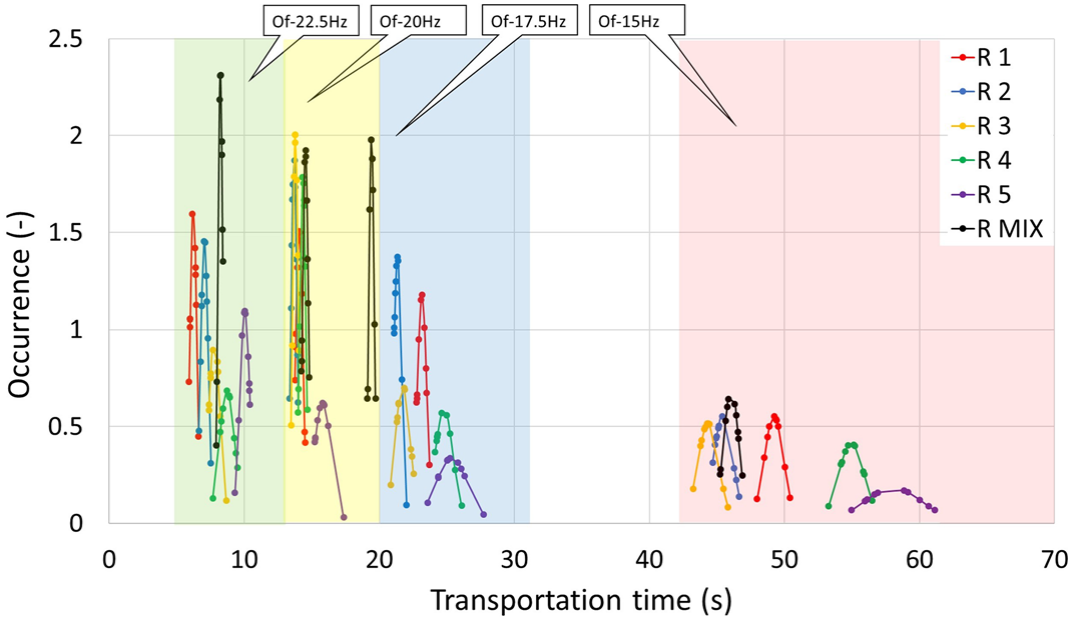
The transport time of the particles in the transport of the rods depends on the different frequencies of the transport distance.

The measurement of the transport speed and the associated measurement of the time it takes for the test material to travel the specified distance was carried out on real devices for the same length (580 mm), same volume of material (800 ml) and for the frequencies of the vibrating conveyor: 15, 17.5, 20 and 22.5 Hz. Data from Table 7 can be divided into two parts. For pellets marked with P(1–5), P MIX and CP, see the graphical display in Fig. 21. For wooden rods marked with R(1–5) and R MIX, see the graphical display in Fig. 22. Table 7 shows that the time it takes for a material to travel a given distance decreases with frequency. Also, changing the length of each group affects the time it travels a given distance; for P(1–5) pellets, the time increases with increasing length. This is not the case with R(1–5) wooden rods at low frequencies. But for higher frequencies, wooden rods behave just like pellets.

**Table 7:** Feed time depending on the oscillation frequency.

	Of 15 (Hz)		Of 17.5 (Hz)		Of 20 (Hz)		Of 22.5 (Hz)	
	Time	σ_sd_ (s)	Time	σ_sd_ (s)	Time	σ_sd_ (s)	Time	σ_sd_ (s)
P1	40.62	0.81	20.18	0.38	13.49	0.24	7.14	0.31
P2	41.63	0.81	20.36	0.32	13.51	0.37	7.59	0.31
P3	41.57	1.24	20.17	0.54	13.81	0.24	8.13	0.21
P4	43.33	0.99	21.09	0.63	13.85	0.43	8.59	0.27
P5	46.58	1.05	21.42	0.42	14.65	0.35	8.41	0.22
P MIX	42.27	0.76	19.88	0.26	14.07	0.25	7.50	0.27
CP	106.70	8.27	28.27	0.97	15.36	0.77	10.53	0.68
R1	49.20	0.72	23.14	0.34	14.07	0.26	6.22	0.24
R2	45.44	0.72	21.34	0.29	13.68	0.21	7.05	0.27
R3	44.34	0.77	21.73	0.56	13.80	0.20	7.79	0.42
R4	54.94	0.96	24.78	0.68	14.33	0.22	8.72	0.58
R5	58.03	2.18	25.35	1.18	15.79	0.64	10.03	0.36
R MIX	46.05	0.58	19.42	0.20	14.52	0.21	8.24	0.17

Figure 21 reveals that the CP sample shows the worst times at 15 Hz. As the frequency increases, that difference shrinks. However, it is still evident that the CP sample shows the worst results. From this perspective, it is clear that by pelleting the CP sample, the cost of transporting it will be reduced.

Figure 21 shows that the CP sample displays the worst stability at a frequency of 15 Hz. With increasing frequency, the sample stabilises. However, the variance values are still consistent with other samples in the 15 Hz vibration range. The P(1–5) and P MIX samples are stabilised with increasing frequency, which corresponds to the measured time of movement depending on the frequency of vibration (Table 7).

For a sample of wooden rods (Fig. 22), the R MIX sample shows the best stability across all frequencies, as opposed to the R5 sample, which shows the worst stability. For wooden rods, the best vibration frequency stability is 20 Hz, which corresponds to the measured data from Table 7.

frequencies, as opposed to the R5 sample, which shows the worst stability. For wooden rods, the best vibration frequency stability is 20 Hz, which corresponds to the measured data from Table 7.

## Conclusions

The work presents the results of comparing the mechanical-physical properties of spruce pellets, crushed pellets and wooden rods. The pellet and wooden rod samples were divided into 5 groups by length. In addition, a detailed analysis of the transport behaviour of these particles was carried out on a test vibrating conveyor. In the first phase, the basic parameters such as internal friction, static angle of repose, dynamic angle of repose, particle size distribution and particle shape were determined. The values of the named properties are important for predicting the behaviour of the tested material during transport processes and also as input parameters into DEM simulations. A vibration device was used to analyse the pellet transport process in its entirety. It is a multi-function device, and all its settings are published in the patent: Method of modelling mechanical processes of bulk materials and device for making the same^19^.

For the purpose of determining the influence of mechanical-physical properties on vibration transport, the possibility of changing the speed of particle displacement was realised by changing amplitude parameters and the frequency of vibrations. From the measured values of particle size distribution, internal friction and angle of repose are illustrative differences between samples of different lengths as well as the CP sample against other samples. All these parameters show that the cylindrical shape of the pellets makes it possible to reduce friction or resistances and thus also the energy performance of transport, or storage. However, transport and storage machines must be designed to prevent material degradation along the route. This would increase the friction and resistance values, in extremis to the measured sample values of CP. For the effective internal friction angle (AIFE) values, the cylindrical particles were found to have on average a smaller effective angle of internal friction of 9.9° than the sample of CP crushed pellets and angle of repose (AOR) of 10.2° less than the CP. This difference is sufficient to ensure that the pellets are not in the zone of cohesive materials, as is the CP sample. Differences can also be seen from particle size distribution results, with CP crushed pellets showing a wide distribution and small particle size. This combination of mechanical-physical properties is disadvantageous compared to pellets for vibratory transport in low layers caused by the 800 ml volume used for all samples. The crushed pellet sample behaves in this case as an aerated powder, the particles of which are light, have dampening effects in the trough bouncing and thus have longer times in relation to the cylindrical shapes (P and R) by about half, which proves a measured value of 106.7 s over a distance of 580 mm at a frequency of 15 Hz. When increasing speed by the rotating of imbalanced wheels, this transport time difference on the test vibrating conveyor—the CP sample versus the pellets—decreases.

In order to also test particles that do not break, crush or have aligned edges as part of measurements or transport, oak poles have been cut into certain lengths. Again, they were divided into groups by length (R1–R5). These created particles exhibited a lower angle of internal friction, precisely because of the properties listed, such as their shape. During vibratory transport, it was found that the excessive perfection of the particle, such as when the ends of the rod particles are aligned vs the ends of spruce pellets having burrs, causes the particles in the low conveyed layer to not sink into each other and, because of these characteristics, to have a greater bounce at the lower vibration frequencies. The boundary between the chaotic bouncing of particles and directed bouncing is already noticeable at the frequency of rotation of the imbalanced conveyor wheels at 20 Hz versus pellets, where this boundary is at 22.5 Hz. From a comparison of pellet and rod measurements, it can also be observed that as the cylindrical particle mass increases, this boundary moves towards higher rates of rotation of the imbalanced wheels of the engine. Since the individual particles also interact with each other in the bounce, there is no possibility of chaotic bouncing to the sides, or even backwards, but they do move in a mass in the direction of the transport.

Within the vibration machine setting, the process preferred for vibration transport matters. In this case, the frequencies up to 17.5 Hz for the rods and up to 20 Hz for the pellets, for the rapid transport process. If a sorting operation is preferred, it is necessary to move above 22.5 Hz for a given amount of material in the area. And if the goal is to do both operations at once, it is recommended to move between those values. This marks the boundary between chaotic (unstable) and directional (stable) particle movement (bouncing).

As it flows around the passive elements, it can be observed that if the vibrations are in a (stable) region, it is possible to base initial predictions on the shape of the particles. Based on this particle information, it can then be predicted whether or not it will pass through the given barrier when vibrating transport at the set vibrations. Again, a compromise is being sought. An example might be that it is desirable for a material to easily flow around a given obstacle but not skip over it or scatter around it. If there is an impediment to the arch, the relationships that are generally used to calculate the storage concerning the size of the opening to the particle apply. Illustratively, in this case, the principle of the gravitational displacement of the material in the vertical storage at discharge can be replaced by the vibration displacement in the laid storage, where the disturbances of the arch can also be seen. In this case too, as with vertical storage, the vault is disrupted, for example, by adding vibration members to the hopper to disrupt the static state of the arch by vibration dynamics. The same is true of an arch in vibrating transport, in an obstacle that resembles an out-hopper from storage. This state can be disturbed by the greater dynamics of the bounce, i.e. when a particle is able to rotate differently relative to other particles and thereby disrupt the resulting arch. This can be seen with the R5 rod sample. At a frequency of 15 Hz on a passive element, arches are formed, but not with higher frequencies.

If the basic measured pellet parameters are entered into the DEM simulation method, it is evident that the result differs from reality. The inaccuracy is due to the fact that the simulation did not have a detailed validation of the correction on the actual restitution coefficient. Such a result from the simulation method is sufficient at an early stage in the development of new devices. However, if the aim is to find a boundary between the directional pattern and random particle bounce, refinement of the simulation by detailed validation^26^, which is performed directly on the vibration device is a necessary part of the correct simulation calculation.

Within Industry 4.0, the standard is to seek a compromise in device settings by, for example, automatically controlling the vibrations of a vibrating conveyor, precisely when using a given device for multiple processes at once, namely sorting and transport.

## Patents

The measurement used a device that was pantented with the title: Validation device and method of measuring static and dynamic angle of discharge. Document number—306123. This device enables the static and dynamic angle of repose piles to be measured in real terms and also by using a 3D model of the device. It addresses the implementation of control and simulation experiments that affect processes in the area of the transport and storage of bulk materials. The solution uses the DEM (Discrete Element Method) method. Based on measured values of the mechanical-physical properties of the real material, programming in EDEM software creates a real mixture for dynamic simulation. The necessary optimisation parameters are obtained by measurement on a real prototype.

Furthermore, the measurement was made using a device that was patented with the title: Method of modelling mechanical processes of bulk materials and device for making the same. Document number—305194. The invention concerns the performing of control and simulation experiments that affect transport by vibrating conveyors.
